# Assessing Fall Risk Factors in Adults 65+ Living in Rhode Island, Massachusetts and New Hampshire

**DOI:** 10.1093/geroni/igaf122.3454

**Published:** 2025-12-31

**Authors:** Nachalie Rodriguez-Cruz, Sophia Casale, Qinglin Gao, Calvin Tran, Elizabeth Dugan

**Affiliations:** University of Massachusetts Boston, Boston, Massachusetts, United States; University of Massachusetts Boston, Red Bank, New Jersey, United States; University of Massachusetts Boston, Boston, Massachusetts, United States; University of Massachusetts Boston, Boston, Massachusetts, United States; University of Massachusetts Boston, Boston, Massachusetts, United States

## Abstract

Falls are one of the leading causes of injury and death for older adults 65+. A number of risk factors for or consequences of falls include hip fractures, osteoporosis, and difficulty with ambulation. This study aims to describe/contrast disparities in community rates of hip fractures, osteoporosis, and ambulatory difficulty in adults 65+ in Massachusetts, Rhode Island, and New Hampshire. This study utilizes data from the 2025 Healthy Aging Data Reports (healthyagingdatareports.org). The hip fracture and osteoporosis rates were calculated from Medicare fee-for-service data of adults 65 + (2020-2021), and ambulation rate difficulties were calculated from American Community Survey (2018-2022). Results showed that state rates for osteoporosis were MA (20.1%) [13.58; 28.6%], RI (18.9%) [13.0; 24.3%], and NH (16.3%) [9.9; 25.7%]. Difficulty with ambulation state rates were MA (18.6%) [6.12; 41.3%], RI (18.7%) [5.77; 27.9%], and NH (16.7%) [0; 42.9%]. Hip fracture state rates were MA (3.20%) [1.53; 4.59%], RI (3.09%) [2.00; 4.07%], and NH (2.79%) [2.04; 3.99%]. A trend was observed across small towns in MA experiencing the greatest rates of osteoporosis (Lenox, 28.6%; Brookline, 27.9%). New Hampshire community and state rates of hip fractures and osteoporosis were lower than MA and RI. The rates of ambulatory difficulty were lower in the southern, wealthier areas of RI. Understanding community rates of the indicators can identify areas underserved and in need of programs or services. Awareness of the disparities across states and within states can help fall prevention, physical activity programming, and hospitals assess the risks individuals within their community experience.

